# Long-term gynecological complications after conservative treatment of placenta accreta spectrum

**DOI:** 10.3389/fmed.2022.992215

**Published:** 2022-10-28

**Authors:** Shmuel Herzberg, Yossef Ezra, Rani Haj Yahya, Carolyn F. Weiniger, Hila Hochler, Doron Kabiri

**Affiliations:** ^1^Department of Obstetrics and Gynecology, Faculty of Medicine, Hadassah Medical Center, Hebrew University of Jerusalem, Jerusalem, Israel; ^2^Division of Anesthesiology, Critical Care and Pain, Tel Aviv Medical Center, Tel Aviv, Israel

**Keywords:** conservative treatment, gynecological complications, fertility, invasive placentation, placenta accreta spectrum

## Abstract

**Objective:**

To examine the association between conservative treatment for PAS (placenta accreta spectrum) and subsequent gynecological and fertility complications.

**Methods:**

All women who underwent conservative treatment for PAS between January 1990 and December 2000 were included in this retrospective cohort study conducted in a tertiary teaching hospital. Gynecological and fertility complications experienced after the index delivery were collected from the medical records and telephone questionnaires. This data was compared to an age and parity-matched control group of women without PAS.

**Results:**

The study group included 134 women with PAS managed conservatively and 134 controls with normal deliveries matched by parity and age. Women in the PAS group required significantly more postpartum operative procedures such as hysteroscopy or D&C (OR = 6.6; 95%CI: 3.36–13.28; *P* = <0.001). Following the index delivery, there were 345 pregnancies among 107 women who attempted conception following conservative treatment for PAS vs. 339 pregnancies among 105 women who attempted conception in the control group. Among women who attempted conception following conservative treatment for PAS 99 (92.5%) delivered live newborns (a total of 280 deliveries) vs. 94 (89.5%) in the control group, (a total of 270 live newborns, *p* = 0.21). The need for fertility treatments was not different between the two groups (OR = 1.22; 95%CI: 0.51–2.93; *P* = 0.66).

**Conclusion:**

After conservative treatment for PAS, significantly more women required complementary procedures due to retained placenta and/or heavy vaginal bleeding. There was no evidence of fertility impairment in women post-conservative treatment for PAS.

## Synopsis

Following conservative treatment for invasive placentation women required more additional procedures such as hysteroscopy or dilatation and curettage due to retained placental tissue and heavy vaginal bleeding. There was no evidence of fertility impairment in this population.

## Introduction

Placenta accreta spectrum (PAS) is a major obstetric complication with rising incidence (1–9) caused by abnormal uterine placental attachment due to the absence of the decidua basalis (10). PAS is a general term encompassing abnormal placentation with varying degrees of involvement (total, partial or focal) and levels of invasion (placenta accreta, increta, and percreta) (10). Recent guidelines (11) suggest a planned cesarean–hysterectomy for cases with high suspicion of PAS, thus, avoiding the risk **of** complication (e.g., hemorrhage) (12).

We previously reported obstetrical outcomes following conservative treatment for PAS using an extirpative technique (13). Similar to other studies we found a higher risk of postpartum hemorrhage and recurrent PAS in subsequent pregnancies. However, when PAS is diagnosed yet uterine conservation was achieved, women may attempt future pregnancies (14). There is a paucity of data regarding other parameters such as quality of life, impact on fertility, and gynecological outcomes after this conservative management. Prior publications of case reports and case series focused on pregnancy outcomes but did not examine other gynecologic problems and subsequent fertility outcomes (15–18).

In this retrospective observational study, we aimed to compare those who underwent conservative treatment for PAS versus a matched cohort of women who did not have PAS for gynecological complications and subsequent fertility.

## Methods

This retrospective cohort study was performed in the Feto-Maternal unit of Hadassah-Hebrew University Medical Center in Jerusalem, Israel, a tertiary teaching hospital. The Institutional Review Board of Hadassah Medical Organization approved the study (decision number 0263-10-HMO), and verbal consent was obtained from all women during a telephone questionnaire.

Our database is described previously (13, 19) and summarized here briefly. The study group cohort comprised all women who underwent conservative treatment for PAS between January 1, 1990, and December 31, 2000, who could be contacted and agreed to participate. Women were allocated to the study group when met clinical or histopathological inclusion criteria for PAS.

The common practice in our unit, when there is a substantial risk for PAS in the ultrasound examination, we usually recommend doing an elective cesarean section with a multidisciplinary team to get prepared for massive bleeding and a hysterectomy when needed. However, in other cases when there is an assessment of PAS during the placenta extraction in normal vaginal delivery or cesarean section, we may consider conservative treatment that usually includes manual lysis of the placenta or D&C.

After using the common procedures of manual revision of uterine cavity or D&C we consider another complementary procedure in cases with clinical suspicion due to symptoms or signs of retained placenta parts in the ultra-sound exam and not as a determined procedure.

Conservative management of PAS was defined as the removal of the placenta with uterine preservation.

Demographic characteristics and matching criteria are presented in Appendix 1.

The control group cohort was selected from the labor and delivery unit electronic medical record following this process: each delivery with PAS from the study group was matched to a normal delivery composing the control group, and according to the consecutive chronological order. The study and control group were matched by maternal age, mode of delivery, and previous live births.

Once the study (prior PAS) and control (normal pregnancy and delivery) groups were identified, the hospital medical records and the Ministry of Health Central Bureau of Statistics data were reviewed to obtain information about obstetrics, fertility, and gynecological parameters until 2010. In addition, we conducted a complementary telephone questionnaire to obtain information regarding complications that appeared after the index delivery (e.g., postpartum operative intervention, the need for fertility treatments, etc.) (Appendix 2).

### Outcomes

The primary outcome measure was unusual postpartum vaginal bleeding that required operative interventions. The secondary outcomes were menstrual cycle irregularity, gynecologic clinic visit frequency, and the need for fertility treatments.

Statistical analysis was performed using SPSS (SPSS Inc., Chicago, IL, USA). Continuous parametric variables are presented using mean SD, and the difference between the two groups was assessed using the student *t*-test. Categorical variables are presented as count (percentage), and the differences between the study and control group for each of the categorical variables were analyzed using χ2 or Fischer exact test. Odds ratios (ORs) were calculated using a multivariable logistic regression model and are presented with 95% confidence intervals. A *p* < 0.05 was considered statistically significant. Missing variables were not imputed.

## Results

Over the ten-year study period, there were 34,450 deliveries at Hadassah-Hebrew University Medical Center, and 260 deliveries had prior PAS and met the study group inclusion criteria. Ninety-nine (38.1%) women were lost to follow-up, 5 (1.9%) women lacked a matched control, and 22 (8.5%) women declined participation. Thus, the final study group included 134 women following conservative treatment for PAS and 134 control women. The groups were similar for demographic and obstetrical parameters.

More women following conservative treatment for PAS reported an unusual vaginal bleeding during the postpartum period (OR = 2.96 95% CI 1.25–7.00; P = 0.01). Additionally, these women required significantly more postpartum operative procedures such as hysteroscopy or “dilation and curettage” secondary to abnormal uterine bleeding (40.8 vs. 9.4%; OR = 6.6 [95%CI = 3.36–13.28], *P* = <0.0001). There were no differences between women following conservative treatment for PAS vs. the control group for menstrual cycle frequency and gynecologic clinic visits in the subsequent years (Table 1).

**Table 1:** Questionnaire responses following the index delivery for women following conservative treatment for placenta accreta spectrum vs. age-parity matched controls.

**Characteristics**	**Study group (*n* = 134)**	**Control group (*n* = 134)**	**OR (95% CI)**	***P* value**
Strong pain after delivery	18 (14.40%)	9 (6.98%)	2.24 (0.97–5.20)	0.06
Unusual bleeding after delivery	20(16.26%)	8 (6.15%)	2.96 (1.25–7.00)	0.01
Dilatation and Curettage (D&C) or hysteroscopy	53(40.77%)	12 (9.38%)	6.6 (3.36–13.28)	<0.0001
Increase in the number of gynecologic clinic visits	7 (5.22%)	5 (3.79%)	1.4 (0.43–4.52)	0.57
Change in the frequency of the menstrual cycle	14 (10.61%)	9 (6.92%)	1.59 (0.66–3.83)	0.29

Values are given as a number (percentage) unless stated otherwise.

Following the index delivery until 2010, there were 345 pregnancies among 107 women who attempted conception following conservative treatment for PAS vs. 339 pregnancies among 105 women who attempted conception in the control group. Many women had more than one pregnancy. Among women who attempted conception following conservative treatment for PAS 99 (92.5%) delivered live newborns (a total of 280 deliveries) vs. 94 (89.5%) in the control group, (a total of 270 live newborns, *p* = 0.21). There were no stillbirths in either group. The proportion of term deliveries, preterm deliveries, miscarriages, and ectopic pregnancies was similar in both groups (Table 2). The number of women who complained about strong pain after the index delivery was not significantly different between the groups (OR = 2.24; 95% CI 0.97–5.20; *p* = 0.06).

**Table 2:** Pregnancy outcomes following the index delivery for women following conservative treatment for placenta accreta spectrum vs. age-parity matched controls.

**Characteristics**	**Pregnancies in the study group (*n* = 345)**	**Pregnancies in the control group (*n* = 339)**	**OR (95% CI)**	***P*-value**
Term-delivery	267 (77.39%)	252 (74.33%)	1.18 (0.83–1.68)	0.35
Pre-term delivery	13 (3.77%)	19 (5.60%)	0.66 (0.32–1.36)	0.26
Miscarriage	57 (16.52%)	62 (18.29%)	0.88 (0.59–1.31)	0.54
Ectopic pregnancy	3 (0.87%)	3 (0.88%)	0.98 (0.20–4.90)	0.98
Termination of pregnancy	5 (1.45%)	3 (0.85%)	1.65 (0.39–6.94)	0.50

Values are given as a number (percentage) unless stated otherwise.

Following the index delivery, the subsequent need for fertility treatments was similar in both groups (OR = 1.22 95% CI 0.51–2.93; *p* = 0.66) (Table 3).

**Table 3:** Fertility treatments following the index delivery for women following conservative treatment for placenta accreta spectrum vs. age-parity matched controls.

	**Study group (*n* = 134)**	**Control group (*n* = 134)**	**OR (95% CI)**	***P*-value**
Ovulation induction	7 (5.30%)	7 (5.30%)	NS	NS
IVF	5 (3.79%)	3 (2.27%)	1.22 (0.51–2.93)	0.66

Values are given as a number (percentage) unless stated otherwise. NS = Non-Significant.

## Discussion

The main finding of the study is that ~40% of women who were treated conservatively for PAS required complementary procedures (i.e., hysteroscopy or dilatation and curettage), due to residual placenta and vaginal bleeding in the puerperium period. This finding emphasizes the importance of close monitoring and prompt intervention in the post-partum period in women who undergo conservative treatment for abnormal placentation.

Due to the high risk for intervention in this population, monitoring should include careful assessment of abnormal uterine bleeding and a sonographic evaluation upon necessity. Numerous studies have demonstrated the utility of postpartum ultra-sound in evaluating residual trophoblastic tissue (20–22). It should be noted that although ultrasound has multiple advantages in the evaluation of residual trophoblastic tissue, it has high false-positive results (23). By using a combination of clinical judgment and sonographic imaging, an accurate diagnosis can be made (24).

When residual trophoblastic tissue is suspected, the gold standard for evaluation is diagnostic hysteroscopy followed by removal of the residua by operative hysteroscopy (25, 26).

The fertility potential after conservative treatment for PAS is unknown. One theory considers these women to be at increased risk for unsuccessful embryo implantation due to abnormal placentation in a prior pregnancy. However previous studies on conservative treatment in cases of PAS found no adverse effect on fertility. Sentilhes et al. reported a retrospective study on women with a history of conservative management for PAS in France. Among 91 women, 9% had severe intrauterine synechiae and were amenorrheic; 30% desired more children; 24 women conceived 34 pregnancies, and 21 deliveries were resulting in healthy babies. The authors concluded that successful conservative treatment for abnormal placentation does not appear to hinder subsequent fertility (15). In our study, the fertility potential of women who underwent conservative treatment in cases of PAS was not affected in comparison to the control group.

This study addresses important issues regarding fertility and gynecological outcomes following conservative treatment for PAS. Additionally, our study provides important information for counseling women regarding their fertility potential and gynecological follow-up care.

In conclusion, approximately 40% of women undergoing conservative treatment for PAS require complementary procedures for uterine emptying, but future fertility potential is not affected.

## Data availability statement

The raw data supporting the conclusions of this article will be made available by the authors, without undue reservation.

## Ethics statement

The studies involving human participants were reviewed and the Institutional Review Board of Hadassah Medical Organization approved the study (Decision Number 0263-10-HMO), and verbal consent was obtained from all women during a telephone questionnaire. Written informed consent for participation was not required for this study in accordance with the national legislation and the institutional requirements.

## Author contributions

SH, YE, and DK are the primary authors of this article. SH, YE, RH, HH, and DK developed the original design, conducted the study, and contributed to the writing of the final version of the article. All authors fulfilled the definition of authorship, contributed to the article, and approved the submitted version.

## Conflict of interest

The authors declare that the research was conducted in the absence of any commercial or financial relationships that could be construed as a potential conflict of interest.

## Publisher's note

All claims expressed in this article are solely those of the authors and do not necessarily represent those of their affiliated organizations, or those of the publisher, the editors and the reviewers. Any product that may be evaluated in this article, or claim that may be made by its manufacturer, is not guaranteed or endorsed by the publisher.
