# Supplementary material for: Long-term gynecological complications after conservative treatment of placenta accreta spectrum
Source: Front Med (Lausanne). 2022 Oct 28;9:992215. doi: 10.3389/fmed.2022.992215 (PMC9650034; doi:10.3389/fmed.2022.992215)
Supplement: Supplementary file 1 [file Data_Sheet_1.PDF]

Table 1

Matching criteria between the study and control groups

	**Study group (N=134)**	**Control group (N=134)**	***P***
Maternal age:			0.99
- 18-24	30 (22.4%)	31 (23.1%)	
- 25-29	34 (25.4%)	35 (26.1%)	
- 30-34	41 (30.6%)	40 (29.9%)	
- 35-39	24 (17.9%)	23 (17.2%)	
- >40	5 (3.7%)	5 (3.7%)	
Mode of delivery			1.00
- Cesarean delivery	3 (2.2%)	3 (2.2%)	
- Vaginal Delivery	131 (97.8%)	131 (97.8%)	
Previous live births			0.90
- 0	36 (26.9%)	37 (27.6%)	
- 1	41 (30.6%)	41 (30.6%)	
- 2	24 (17.9%)	20 (14.9%)	
- 3-5	18 (13.4%)	23 (17.2%)	
- 6 or more	15 (11.2%)	13 (9.7%)	

Table 2

Demographic and obstetric characteristics at the elected delivery for women included in the study and control groups

	Study group (N=134)	Control group (N=134)	*P*
**Demographic characteristics**			
Maternal age	30.58 ± 5.647 [21-44]	30.4 ± 5.774 [20-40]	0.925
Gravidity	2.71 ± 2.99 [0-13]	2.64 ± 2.89 [0-15]	0.879
Parity	1.92 ± 2.22 [0-10]	2.07 ± 2.32 [0-10]	0.456
Abortions	0.71 ± 1.27 [0-9]	0.56 ± 1.09 [0-7]	0.263
Ectopic pregnancy	0.04 ± 0.27 [0-2]	0.01 ± 0.086 [0-1]	0.174
Prior cesarean section	0.08 ± 0.31 [0-3]	0.11 ± 0.276 [0-1]	0.410
Living children	1.95 ± 2.25 [0-10]	1.98 ± 2.33 [0-12]	0.992
Previous placenta accreta	23 (17.2)	4 (3)	<0.001
Previous placenta previa	2 (1.5)	3 (2.2)	0.868
Previous post-partum hemorrhage	11 (8.2)	3 (2.2)	0.037
**Obstetrical characteristics**			
Cesarean delivery	3 (2.2)	3 (2.2)	1.00
Hypertension disorders	11 (8.2)	4 (3)	0.104
Infections	7 (5.2)	4 (3)	0.372

Data are expressed as mean ± SD [range], or N (%).
