# Supplementary material for: Long-term gynecological complications after conservative treatment of placenta accreta spectrum
Source: Front Med (Lausanne). 2022 Oct 28;9:992215. doi: 10.3389/fmed.2022.992215 (PMC9650034; doi:10.3389/fmed.2022.992215)
Supplement: Supplementary file 2 [file Data_Sheet_2.PDF]

Follow-up questionnaire – Study group

Name of reviewer: __________ Date______________

Part I - medical records

Demographic data:

Name of the patient: _________________ ID _____________________

Residence_______________-

Smoking - 1.Yes 2. No

Religion - 1. Jewish 2. Muslim 3. Christian 4. Druze 5. Other

Medical history:

Type of delivery (the delivery that was complicated with placenta accreta) - 1. Vaginal delivery 2. Cesarean delivery.

Age at the event -______ Date of birth__________

Number of pregnancies (G) -_____________ Number of births (P)________

Number of abortions (AB) -_____________ Number of ectopic pregnancies (EP) ________

Number of living children (LC)__________

Number of Caesarean section (CS)______

Was there a previous pregnancy with placenta accreta? 1. Yes 2. No 3. Unknown

Was there a previous pregnancy with placenta previa - 1. Yes 2. No 3. Unknown

Was there a post partum hemorrhage before? 1. Yes 2. No

Did the patient have hypertension? 1.During pregnancy(after 20th week). 2.Chronic hypertension(before 20th week) 3.No 4. Unknown

Total number of hospitalization days: ___.

ICU hospitalization days: ___.

Sex of the newborn - 1. Male 2. Female.

Birth weight -___________ grams

Apgar: 1 minute _______ 5 minutes ________ 10 minutes _________

If mode of delivery was cesarean:

Cause of surgery - 1. Fetal position 2. Other_______

Was the patient treated with blood transfusion after delivery? 1. Yes 2. No

Placenta accreta details:

Method of diagnosis 1. Before delivery by US. 2. Before delivery by MRI 3. During the delivery.

Treatment - 1. Medical treatment by uterotonic agents 2. Ligation of blood vessels 3. Embolization of blood vessels 4. B-Lynch 5. Hysterectomy 6. Other _____________

Part 2 - Telephone questionnaire

Demographic data:

Name of the patient: _________________ ID_____________________

Residence -_______________ Current age__________

Smoking? 1. Yes 2. No

Religion: 1. Jewish 2. Muslim 3. Christian 4. Druze 5. Other_____________-

Events that occurred after the delivery complicated with placenta accreta:

In this study, we try to find various events that occurred following the delivery complicated with placenta accreta, I would be happy if you agree to answer a few questions:

What was the mode of the delivery: 1. Vaginal delivery 2. Cesarean delivery.

1. Did you have severe pain after the delivery? 1. Yes 2. No 3.I do not remember, Details_____________

2. Did you suffer from unusual bleeding after the delivery (over 6 weeks)? 1. Yes 2. No 3. I do not remember

3. Have you undergone any gynecological surgery following the delivery / CD? Operative hysteroscopy? 1. Yes 2. No

D&C? 1. Yes 2. No

Other, Details______________

From the delivery until today-

What is the nature of your cycle? 1. Regular cycle - under hormonal therapy 2. Regular cycle - without hormonal therapy 2. Irregular cycle 3. Amenorrhea, lasting _______

Has there been a change in the nature of the menstrual cycle since the delivery?

Did the frequency of visits to a gynecologist clinic increase since the delivery? 1. Yes 2. No

Obstetric follow-up:

Did you try to get pregnant after the delivery(complicated with placenta acrreta)? 1. Yes 2. No.

* If the answer is yes-

1. Did you undergo fertility treatments? 1. No 2. Ovulation induction 3. IVF 4. Other ___________

2. Did you get pregnant after surgery? 1.Yes   2.No

Pregnency no.	Termination of pregnancy	Miscarriage	Ectopic pregnancy	Preterm delivery	Term delivery
1					
2					
3					
4					
5					

Delivery no.	Gestation week	Mode of delivery CS\VD	Birth weight	APGAR Score

Subsequent pregnancies events:

1. Did you have recurrent event of placenta accreta? 1. Yes 2. No 3. I do not remember / I do not know.
2. Did you have placenta previa? Yes 2. No 3. I do not remember / I do not know.
3. Did you have heavy bleeding that required a blood transfusion? 1.Yes 2. No 3. I do not remember / I do not know.
4. Did you have hypertension during the pregnancy or delivery? 1. Yes - after 20th week 2. Yes - chronic hypertension (before 20th week) 3. No 4. I do not know

Thank you for your time, are there other relevant details we did not ask, you think we should know?

____

____

____

Follow-up questionnaire - control group

Name of reviewer: __________ Date______________

Part I - medical record

Demographic data:

Name of the patient: _________________ ID _____________________

Residence_______________-

Smoking - 1.Yes 2. No

Religion - 1. Jewish 2. Muslim 3. Christian 4. Druze 5. Other

Medical history:

Type of delivery (the relevant delivery) - 1. Vaginal delivery 2. Cesarean delivery.

Age at the relevant delivery -______ Date of birth__________

Number of pregnancies (G) -_____________ Number of births (P)________

Number of abortions (AB) -_____________ Number of ectopic pregnancies (EP) ________

Number of living children (LC)__________

Number of Caesarean deliveries (CD)______

Was there a previous pregnancy with placenta accreta? 1. Yes 2. No 3. Unknown

Was there a previous pregnancy with placenta previa - 1. Yes 2. No 3. Unknown

Was there a post partum hemorrhage before? 1. Yes 2. No

Did the patient have hypertension? 1. During pregnancy (after 20th week). 2. Chronic hypertension (before 20th week) 3.No 4. Unknown

Total number of hospitalization days: ___.

ICU hospitalization days: ___.

Sex of the newborn - 1. Male 2. Female.

Birth weight -___________ grams

Apgar: 1 minute _______ 5 minutes ________ 10 minutes _________

If mode of delivery was cesarean:

Cause of surgery - 1. Fetal position 2. Other_______

Was the patient treated with blood transfusion after delivery? 1. Yes 2. No

Part 2 - Telephone questionnaire

Demographic data:

Name of the patient: _________________ ID_____________________

Residence -_______________ Current age__________

Smoking? 1. Yes 2. No

Religion: 1. Jewish 2. Muslim 3. Christian 4. Druze 5. Other_____________-

Events that occurred after the delivery:

In this study, we try to find events that occurred following the delivery in year _____, I would be happy if you agree to answer a few questions:

What was the mode of delivery: 1. Vaginal delivery 2. Cesarean delivery.

1. Did you have severe pain after the delivery? 1. Yes 2. No 3.I do not remember, Details_____________

2. Did you suffer from unusual bleeding after delivery (over 6 weeks)? 1. Yes 2. No 3. I do not remember

3. Have you undergone any gynecological surgery following the delivery / CD?

Operative hysteroscopy? 1. Yes 2. No

D&C? 1. Yes 2. No

Other, Details______________

From the delivery until today-

What is the nature of your cycle? 1. Regular cycle - under hormonal therapy 2. Regular cycle - without hormonal therapy 2. Irregular cycle 3. Amenorrhea, lasting _______ days

Has there been a change in the nature of the menstrual cycle since the delivery?

Did the frequency of visits to a gynecologist clinic increase since the delivery? 1. Yes 2. No

Obstetric follow-up:

Did you try to get pregnant after the relevant delivery? 1. Yes 2. No.

* If the answer is yes-

1. Did you undergo fertility treatments? 1. No 2. Ovulation induction 3. IVF 4. Other ____________

2. Did you get pregnant after surgery? 1.Yes 2.No

Pregnency no.	Termination of pregnancy	Miscarriage	Ectopic pregnancy	Preterm delivery	Term delivery
1					
2					
3					
4					
5					

Delivery no.	Gestation week	Mode of delivery CS\VD	Birth weight	APGAR Score

Subsequent pregnancies events:

1. Did you have placenta accreta? 1. Yes 2. No 3. I do not remember / I do not know.
2. Did you have placenta previa? Yes 2. No 3. I do not remember / I do not know.
3. Did you have heavy bleeding that required a blood transfusion? 1.Yes 2. No 3. I do not remember / I do not know.
4. Did you have hypertension during the pregnancy or delivery? 1. Yes - after 20th week 2. Yes - chronic hypertension (before 20th week) 3. No 4. I do not know

Thank you for your time, are there other relevant details we did not ask, you think we should know?

____

____

____
